# How hunger guides new brain cells to their destination

**DOI:** 10.7554/eLife.109178

**Published:** 2025-10-20

**Authors:** Fang-Shin Nian, Laurent Nguyen

**Affiliations:** 1 Laboratory of Molecular Regulation of Neurogenesis, GIGA Institute, University of Liège Liège Belgium; 2 WELBIO department, WEL Research Institute Wavre Belgium

**Keywords:** Ghrelin, neurogenesis, metabolism, blood flow, migration, Mouse

## Abstract

Blood flow and a hormone called ghrelin help new neurons travel to where they are meant to be in the brain of adult mice.

**Related research article** Ogino T, Saito A, Sawada M, Takemura S, Hara Y, Yoshimura K, Nagase J, Kawase H, Sato T, Inada H, Herranz-Pérez V, Mukouyama Y, Ema M, García-Verdugo JM, Nabekura J, Sawamoto K. 2024. Neuronal migration depends on blood flow in the adult brain. *eLife* **13**:RP99502. doi: 10.7554/eLife.99502.

The migration of neurons is a fundamental process that shapes brain circuits by ensuring that new neurons reach their appropriate destinations. This phenomenon occurs across several developmental stages, from the formation of brain networks in the embryonic brain to the continuous addition of new neurons in the adult brain.

Blood vessels have long been recognized as important guides for migrating cells, serving as pathways and sources of signals. During embryogenesis, for example, vascular cues can attract distinct cell types to vessel-rich regions, where interactions with blood vessels help interneurons to navigate toward the cortex (Lepiemme et al., 2022). Neuronal migration persists in specialized areas in the adult brain: new brain cells generated in a region of the brain called the subventricular zone travel through the rostral migratory stream to the olfactory bulb, frequently aligning with blood vessels along the way.

While these studies highlight the versatility of the vasculature as a guiding substrate, an important question remained: can differences in blood flow influence cell migration? Now, in eLife, Kazunobu Sawamoto (Nagoya City University and the National Institute for Physiological Sciences) and colleagues – including Takashi Ogino and Akari Saito as joint first authors – report that neurons preferentially migrate near vessels with abundant flow (Ogino et al., 2024).

The researchers – who are based at institutes in Japan, Spain and the US – investigated the influence of blood flow on the migration of newly generated neurons along blood vessels in the rostral migratory stream and olfactory bulb of adult mice. Using three-dimensional imaging, they were able to track the migration pattern of newly generated neurons in mice that were 6–12 weeks old. This revealed that neurons accumulated near blood vessels with a high flow rate. Experimentally decreasing the flow either slowed or stopped their migration.

Ogino et al. also identified ghrelin – a hormone that rises during fasting – as a factor that mediates the effect of flow rate on migration. Ghrelin can cross the vascular wall into the brain and has been shown to act directly on migrating neurons through its receptor, GHSR1a. By introducing fluorescently labelled ghrelin into the bloodstream of the mice, the researchers were able to show that ghrelin accumulated in the rostral migratory stream and olfactory bulb. Moreover, it increased the speed of the neurons and the distance they travelled. In contrast, disabling Ghsr1a abolished these effects. Together, these results confirm that ghrelin is a critical mediator of blood-vessel-guided neuronal migration (Figure 1).

**Figure 1.**
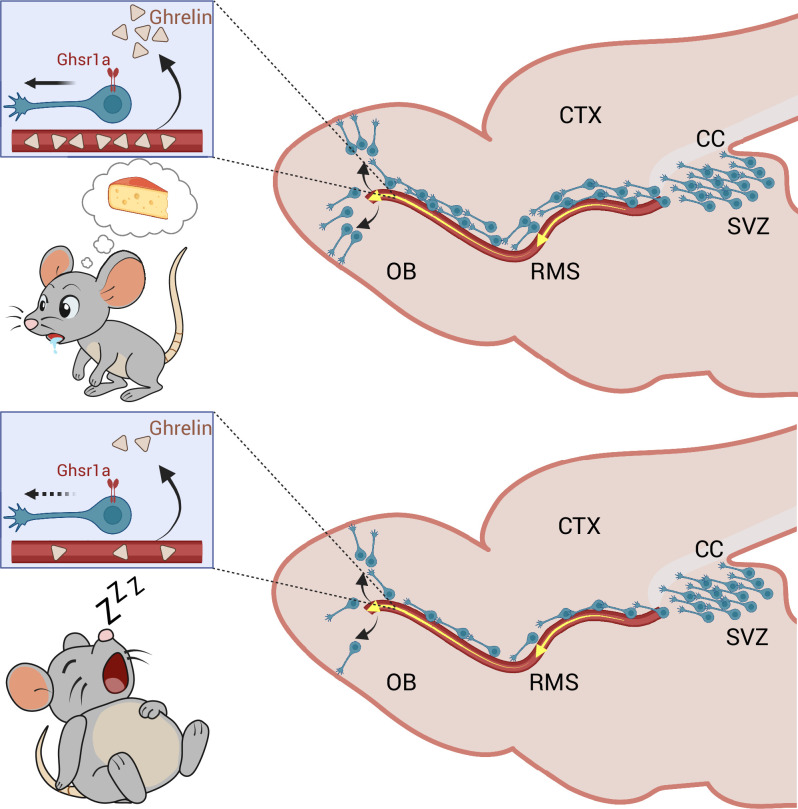
The impact of blood flow and hunger hormones on the migration of neurons. The production of a hormone called ghrelin increases when an animal experiences hunger. The hormone is transported to the brain through the bloodstream, where it crosses the vascular wall and enters a region of the brain called the parenchyma. Ogino et al. found that neurons recognize ghrelin through the receptor Ghsr1a, which promotes the migration of new neurons from the subventricular zone (SVZ) to the olfactory bulb (OB) via the rostral migratory stream (RMS). **Top:** In hungry mice, ghrelin expression is increased, which results in more neurons reaching the OB. **Bottom:** Ghrelin levels are lower in mice that have been fed, so although the number of newborn neurons in the SVZ is comparable to that in hungry mice, fewer of these neurons migrate to the OB.CTX: cerebral cortex; CC: corpus callosum. Created using BioRender.com.

Physiological experiments supported ghrelin’s role in cell migration. Calorie restriction in mice – which coincides with a rise in ghrelin – increased the proportion of neurons in the adult olfactory bulb. This effect was lost in mice without Ghsr1a, indicating that ghrelin signaling is required for the fasting-induced increase in cell migration. Notably, Ogino et al. also demonstrated blood-flow-dependent migration in a primate, the marmoset, highlighting that this mechanism is important across different species. In marmosets, new neurons were preferentially located along arterioles – vessels associated with higher flow – suggesting that this mechanism is important across different species.

The findings of Ogino et al. reveal a previously unrecognized role of blood flow and metabolic hormones in shaping neuronal migration, expanding our view of how systemic physiology shapes brain plasticity. Uncovering ghrelin as a critical mediator opens the door to exploring other blood-borne factors that may guide neurons in both health and disease.

These results raise broader questions about whether vascular dynamics and circulating factors could also be applied in pathological contexts. For example, after brain injury or in neurodegenerative diseases, blood vessels remodel and release a variety of signals that may guide neural progenitor cells toward damaged regions (Kojima et al., 2010). Identifying additional blood-derived mediators could therefore inform strategies to enhance the replacement and repair of neurons, extending the relevance of this work well beyond metabolic regulation.

Moreover, the findings of Ogino et al. suggest that blood-flow-dependent neuronal migration is conserved across rodents and primates. Although direct tests of ghrelin signaling were not performed in primates, the high sequence similarity of the ghrelin receptor between marmosets and mice hints at a conserved hormonal mechanism. This highlights the evolutionary relevance of vascular dynamics for neuronal migration and suggests potential implications for the human brain. Future studies will be needed to determine how vascular signals can be applied to promote brain repair, and whether manipulating such pathways could provide new strategies for neurological disorders.
